# Structure of the Phosphatase Domain of the Cell Fate Determinant SpoIIE from *Bacillus subtilis*

**DOI:** 10.1016/j.jmb.2011.11.017

**Published:** 2012-01-13

**Authors:** Vladimir M. Levdikov, Elena V. Blagova, Andrea E. Rawlings, Katie Jameson, James Tunaley, Darren J. Hart, Imrich Barak, Anthony J. Wilkinson

**Affiliations:** 1 Structural Biology Laboratory, Department of Chemistry, University of York, York YO10 5YW, UK; 2 European Molecular Biology Laboratory Grenoble, BP 181, 6 rue Jules Horowitz, 38042 Grenoble Cedex 9, France; 3 Institute of Molecular Biology, Slovak Academy of Sciences, 845 51 Bratislava 45, Slovakia

**Keywords:** SeMet, selenomethionine, PSP, protein serine phosphatase, ESRF, European Synchrotron Radiation Facility, MAD, multiwavelength anomalous dispersion, SpoIIE, phosphatase, crystal structure, sporulation, manganese binding

## Abstract

Sporulation in *Bacillus subtilis* begins with an asymmetric cell division producing two genetically identical cells with different fates. SpoIIE is a membrane protein that localizes to the polar cell division sites where it causes FtsZ to relocate from mid-cell to form polar Z-rings. Following polar septation, SpoIIE establishes compartment-specific gene expression in the smaller forespore cell by dephosphorylating the anti-sigma factor antagonist SpoIIAA, leading to the release of the RNA polymerase sigma factor σ^F^ from an inhibitory complex with the anti-sigma factor SpoIIAB. SpoIIE therefore couples morphological development to differential gene expression. Here, we determined the crystal structure of the phosphatase domain of SpoIIE to 2.6 Å spacing, revealing a domain-swapped dimer. SEC-MALLS (*s*ize-*e*xclusion *c*hromatography with *m*ulti-*a*ngle *l*aser *l*ight *s*cattering) analysis however suggested a monomer as the principal form in solution. A model for the monomer was derived from the domain-swapped dimer in which 2 five-stranded β-sheets are packed against one another and flanked by α-helices in an αββα arrangement reminiscent of other PP2C-type phosphatases. A flap region that controls access of substrates to the active site in other PP2C phosphatases is diminished in SpoIIE, and this observation correlates with the presence of a single manganese ion in the active site of SpoIIE in contrast to the two or three metal ions present in other PP2C enzymes. Mapping of a catalogue of mutational data onto the structure shows a clustering of sites whose point mutation interferes with the proper coupling of asymmetric septum formation to sigma factor activation and identifies a surface involved in intramolecular signaling.

## Graphical abstract

**Graphical Abstract:**

**Figure:**
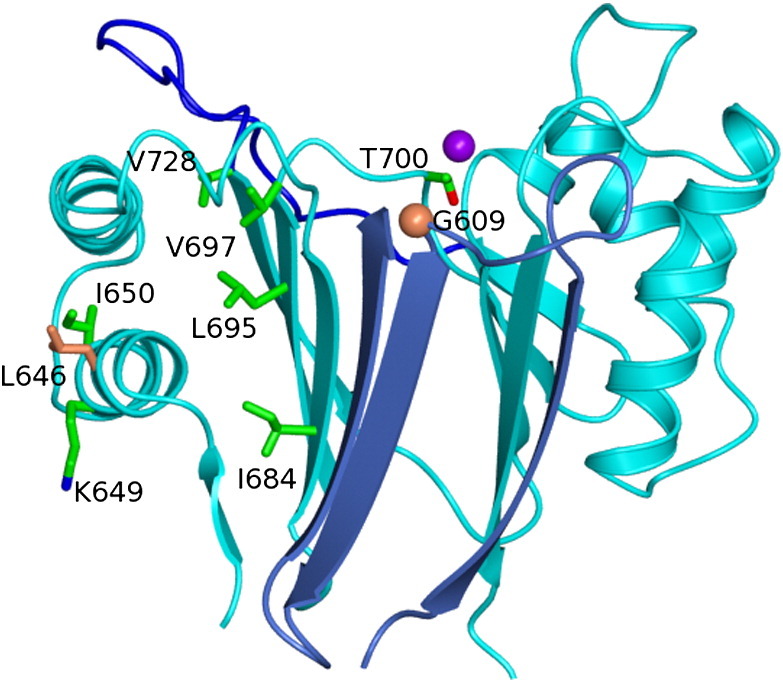

**Highlights:**

► We have determined the crystal structure of the PP2C phosphatase domain of SpoIIE. ► We observe a single bound manganese ion in contrast to the two or three seen in other PP2C enzymes. ► An intramolecular signaling surface is identified by mapping mutational data onto the structure.

## Introduction

The formation of a resistant spore is a remarkable adaptive process undertaken by *Bacillus subtilis* and its relatives under conditions of extreme nutrient limitation.^1^ It begins with an asymmetric cell division giving rise to a larger mother cell and a smaller forespore. The mother cell goes on to engulf the forespore in a process reminiscent of phagocytosis, and the two cells collaborate in the construction of a complex proteinaceous structure, called the coat, around the developing spore. In the final stages, the mother cell undergoes programmed cell death, releasing the mature spore that is robust enough to endure physical and chemical challenges and to survive indefinitely in a state of dormancy. Accompanying these morphological changes is a coordinated developmental program of differential gene expression involving intercellular signaling processes that leads to the sequential activation of the RNA polymerase sigma factors σ^F^ and σ^G^ in the forespore and σ^E^ and σ^K^ in the mother cell.^2,3^

**Fig. 1:**
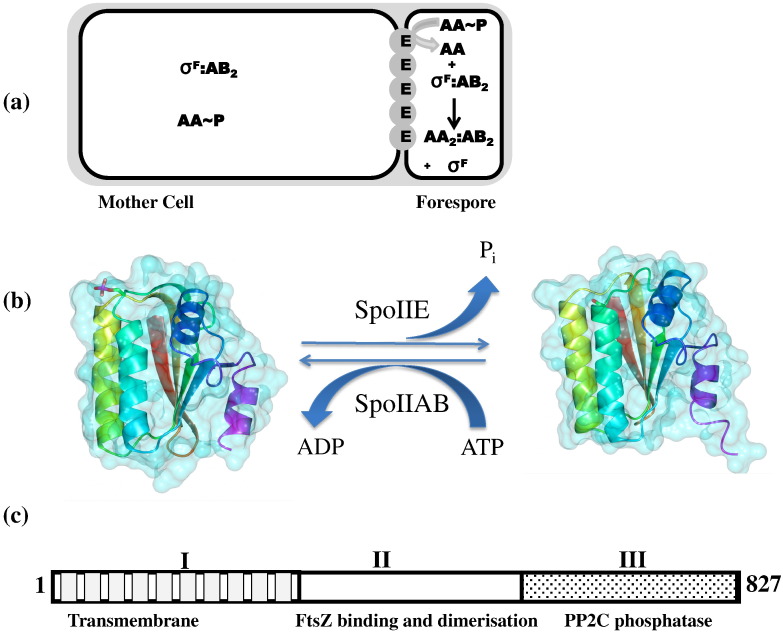
Asymmetric cell division, σ^F^ activation and the role of SpoIIE. (a) In the pre-divisional cell (not shown) and the mother cell following cell division (left), SpoIIAA (AA) is phosphorylated and σ^F^ is in a complex with SpoIIAB (AB_2_). SpoIIE (E) accumulates at the asymmetric septum, a double-membrane structure. In the forespore (right), σ^F^ is free and AA and AB are in complex. The two cells are surrounded by a layer of cell wall (shaded). (b) The structures of SpoIIAA∼ P (left) and SpoIIAA (right) and their interconversion by phosphorylation by SpoIIAB and dephosphorylation by SpoIIE. This and subsequent structure figures were prepared in the program CCP4MG.^50^ (c) The putative three-domain structure of SpoIIE. The limits of the putative FtsZ binding domain are uncertain.

The activation of σ^F^ is a key step in this cascade of gene expression.^4,5^ Although σ^F^ becomes active only in the forespore, it is present in the pre-divisional cell and partitions into both compartments following the asymmetric septation (Fig. 1a). σ^F^ (SpoIIAC) activity is controlled by the interactions of SpoIIAB (an anti-sigma factor and protein kinase), SpoIIAA (an antagonist as well as a substrate of SpoIIAB) and SpoIIE (a septum-localizing protein phosphatase).^6,7^ In the pre-divisional cell and the mother cell, SpoIIAA is phosphorylated and inactivated by SpoIIAB. The latter is therefore available to form a SpoIIAB_2_:σ^F^ complex in which the sigma factor is inactive. In the forespore, the action of SpoIIE leads to the dephosphorylation and activation of SpoIIAA. SpoIIAA can induce the release of σ^F^ from the SpoIIAB_2_:σ^F^ complex, allowing it to bind to core RNA polymerase and direct transcription of forespore-specific genes (Fig. 1a).^8,9^

SpoIIE has a second function in determining the site of formation of the sporulation septum.^10,11^ As occurs during normal cell division, the tubulin-like protein FtsZ forms a ring at mid-cell at the onset of sporulation.^12^ However, the division apparatus is not assembled at this site; instead, the Z-ring migrates from mid-cell on a spiral trajectory to both poles in a process that depends on sporulation-specific overexpression of *ftsAZ* and the presence of SpoIIE.^12^ SpoIIE colocalizes with the polar Z-rings in the form of E-rings. One of the Z/E-rings dissolves, while the other matures into the sporulation septum. Dynamic localization studies using SpoIIE-GFP (*g*reen *f*luorescent *p*rotein) fusions have shown that, following cytokinesis, SpoIIE is released from the septal membrane, transiently becoming distributed throughout the membrane surrounding the forespore.^13^ Subsequently, it relocalizes to the septal membrane as the process of engulfment is initiated. This relocalization is dependent on SpoIIQ, a forespore-specific protein transcribed by RNA polymerase containing σ^F^. These observations point to a third function for SpoIIE following the activation of σ^F^.

The structures of the SpoIIA proteins have been determined, revealing the active and inactive (phosphorylated) forms of SpoIIAA (Fig. 1b)^14,15^ and the interactions of SpoIIAB with σ^F^ and SpoIIAA.^9,16^ In contrast, the structure of SpoIIE and the character of its interactions with SpoIIAA, FtsZ and SpoIIQ are unknown. SpoIIE from *B. subtilis* is an 827-residue protein thought to consist of three domains (Fig. 1c). It has 10 putative membrane-spanning segments at its N-terminus and a PP2C-type phosphatase domain at its C-terminus.^17^ The central domain whose boundaries are uncertain has no resemblance to domains of known structure or function and is conserved only among SpoIIE orthologues. It is involved in interactions with FtsZ and is thought to mediate oligomerization of SpoIIE.^18^ Difficulties in soluble expression of SpoIIE have been an obstacle to biophysical characterization and structural studies. Previously, using a random truncation library approach,^19^ we identified a set of soluble fragments of SpoIIE,^20^ which can be overproduced and characterized. Here, we present the crystal structure of SpoIIE(590–827) encompassing the PP2C phosphatase domain.

## Results

### Protein production and characterization

**Fig. 2:**
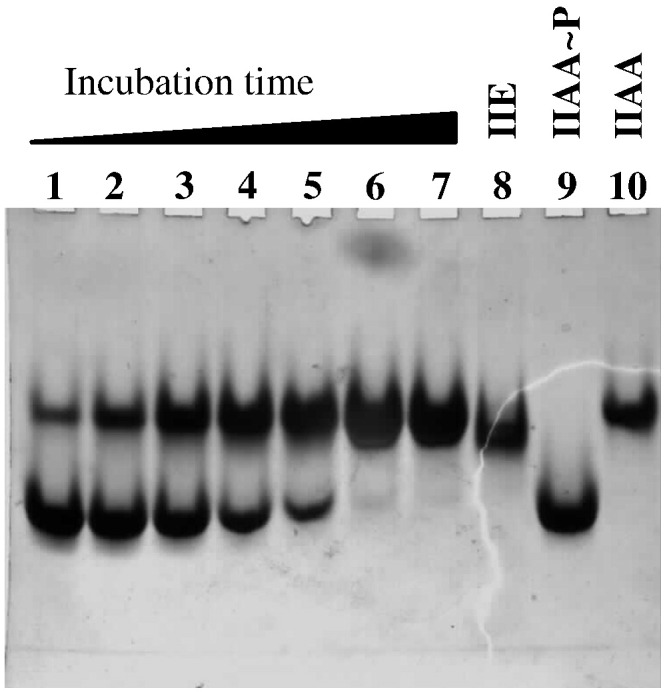
Coomassie-blue-stained non-denaturing polyacrylamide gel demonstrating SpoIIE(590–827) phosphatase activity. SpoIIE(590–827) (50 ng) and SpoIIAA∼P (50 μg) were mixed (a molar ratio of 1:2000), incubated for the times indicated below, and the samples were loaded onto a 7.5% polyacrylamide gel. Electrophoresis was performed at 4 °C for 140 min at 100 V. Lanes 1–7: incubation times of 15, 30, 60, 90, 120, 150 and 180 min . Lane 8, 20 μg SpoIIE(590–827); lane 9, 17 μg SpoIIAA∼ P; lane 10, 17 μg SpoIIAA.

A 228-residue C-terminal fragment encompassing the phosphatase domain of SpoIIE from *B. subtilis* was overproduced in *Escherichia coli* and purified as described in Materials and Methods. Activity assays using SpoIIAA∼ phosphate (SpoIIAA∼ P) from *Bacillus sphaericus* as the substrate and using gel retardation as an assay of reaction progress showed slow turnover and failure of the reaction to proceed to completion.^20^ This could be explained either by the intrinsically low phosphatase activity of the PP2C phosphatase domain in the absence of the upstream protein domains or by the poorer substrate characteristics of the heterologous SpoIIAA∼ P from *B. sphaericus*. For this reason, we prepared the cognate substrate by co-expression of *B. subtilis spoIIAA* and *spoIIAB*. As shown in Fig. 2, SpoIIE(590–827) fully converts the faster migrating *B. subtilis* SpoIIAA∼ P to the slower migrating dephosphorylated form at an enzyme-to-substrate ratio of 1:2000 over a period of 2–3 h. Moreover, the reaction is manganese dependent as evidenced by the persistence of phosphorylated SpoIIAA (i) in the absence of added manganese, (ii) upon the addition of metal chelators such as citrate or (iii) upon replacement of manganese by magnesium (Fig. S1). We conclude that the activity of the phosphatase domain of SpoIIE from *B. subtilis* is ∼ 100-fold higher with the homologous SpoIIAA∼ P substrate.

### Structure solution

To determine the structure of the PP2C phosphatase domain of SpoIIE, we grew crystals of SpoIIE(590–827). The crystals appeared from Tris-buffered sodium citrate solutions at pH 8.5 and belong to the space group *P*6_1_22 with *a* = *b* = 89 Å and *c* = 319 Å. Native data and selenomethionine (SeMet) derivative data at three wavelengths were obtained from these crystals, and the structure was solved by multiwavelength anomalous dispersion (MAD) phasing techniques (Table 1). The coordinates were refined against data extending to 2.6 Å spacing.

**Fig. 3:**
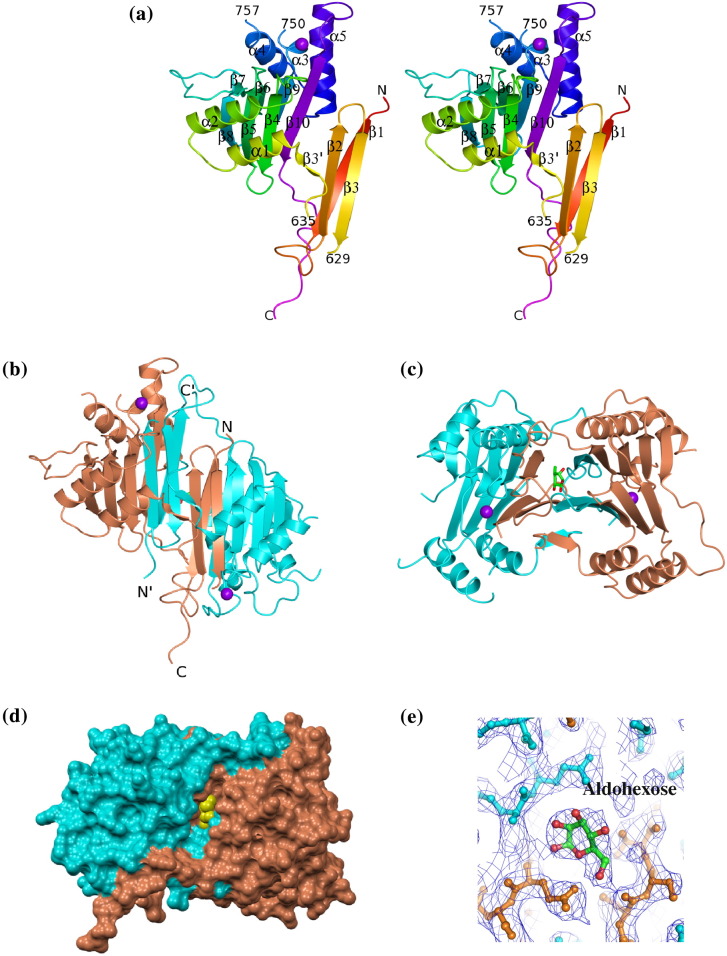
The structure of SpoIIE(590–827). (a) Stereo ribbon representation of the SpoIIE(590–827) phosphatase domain fragment. Chain B is color ramped from its N-terminus (red) to its C-terminus (magenta). The secondary structure elements are labeled, and the positions of breaks in the chain tracing are numbered. Mn^2+^ is represented as a purple ball. The detachment of the β1–β2–β3 segment from the rest of the structure is evident. (b–e) The domain-swapped dimer in approximately orthogonal views (b and c) and in space-filling representation (d). The two subunits are colored cyan and coral, and the chain termini are labeled in (b) and distinguished by the use of an apostrophe ('); Mn^2+^ are shown as purple balls. The more intimate association of the β1–β2–β3 segment with the partner protomer is evident. A cavity is formed at the subunit interface (d), and this is occupied by electron density (e) that cannot be accounted for by protein atoms; an aldohexose sugar has been modeled. As this sugar sits on a pseudo-2-fold axis, two half-occupancy molecules have been modeled, one of which is shown here. The origin of the sugar, if it is a sugar, is unknown. The sugar is shown in yellow space-filling format in (d), in cylinder format colored by atom in (b) and (c) and in ball-and-stick format in (e).

There are two molecules (A and B) of SpoIIE(590–827) in the asymmetric unit of the crystal, giving a solvent content of 63%. The electron density maps define the amino-terminus of both molecules with an Ala–Met sequence inherited from the purification tag visible in chain A. There are gaps in the electron density maps spanning residues 630–634, 672–675, 717–721 and 812–827 of chain A and residues 630–634, 751–756 and 820–827 of chain B, and these parts of the structure are assumed to be disordered. Each chain consists of 10 segments of β-strand and 5 segments of α-helix (Fig. 3a). The overall chain structure comprises a pair of detached subdomains. The first is a small β-sandwich with strand β1 packed against strands β2 and β3; the second is a larger β-sandwich in which a four-stranded β-sheet packs against a three-stranded β-sheet with flanking α-helices. In the course of building into the electron density maps, it became apparent that SpoIIE(590–827) in the crystal is a domain-swapped dimer (Fig. 3b and c) with β-strands 1–3 exchanged between subunits. There is disorder in both chains at residues 630–634 in the linker segment between the third β-strand and the first α-helix. To establish the chain connectivity, we compared the possible distances of separation of the C^α^ atoms of residue 629 from those of residue 635. In the domain-swapped dimer, this distance is 10 Å, while without domain swapping, this distance would be 30 Å, an impossible span for six residues.

As can be seen in Fig. 3b and c, the dimer is characterized by an extensive β-sandwich structure in which a 10-stranded antiparallel β-pleated sheet packs against 2 five-stranded antiparallel β-pleated sheets. The β-sandwich is flanked by α-helices (Fig. 3c). In the dimer, strands 1–3 are diverted from their assembly into intramolecular β-sheets by their reciprocal exchange with the equivalent strands from the partner subunit in the dimer (Fig. S2). It can be seen in Fig. 3c that the chains form a further intermolecular two-stranded β-sheet as they pass from one domain to the other.

At the dimer interface, a pronounced cavity is formed between the edges of the five-stranded β-sheets (Fig. 3d). There is a pseudo-2-fold axis of symmetry running through the cavity whose sides are formed by the β1 strands on each of the two subunits of the domain-swapped dimer. This cavity contained a strong feature in the electron density maps that could not be accounted for by protein atoms. Its size and shape suggested a ring structure, perhaps an aldohexose (Fig. 3e). A xylose in two half-occupancy alternative conformations could be satisfactorily fitted into this density and refined. The presence of such a sugar in the structure is consistent with the observation of species in the mass spectrum of the SpoIIE(590–827) sample with mass differences of 181 Da. The assignment is nevertheless tentative as the origin of this sugar is unknown.

### The PP2C domain is normally a monomer

**Fig. 4:**
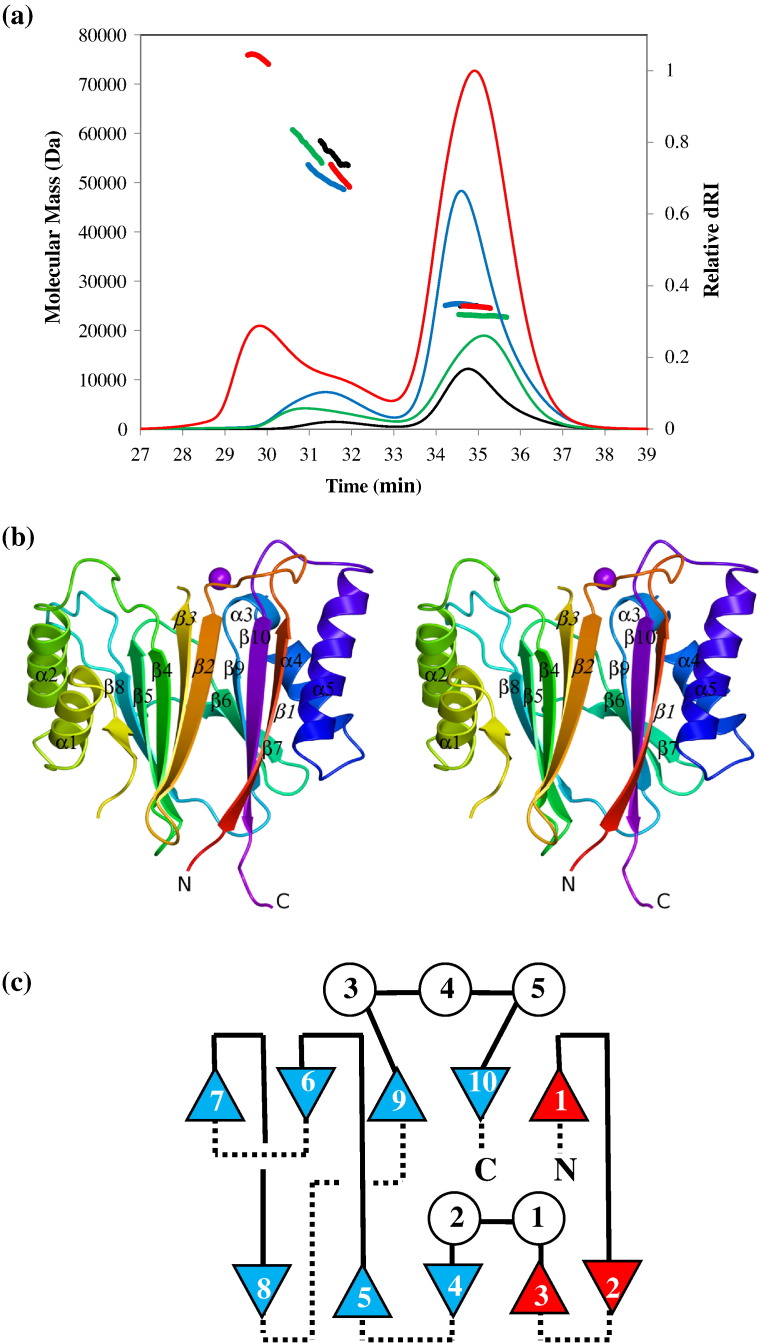
The PP2C domain of SpoIIE is a monomer. (a) SEC-MALLS traces of the molecular mass and differential refractive index (dRI) *versus* time. The thinner lines trace the absorbance at 280 nm of the eluate from a Superdex 10/300 S200 GL column as a function of time. The thicker lines represent the weight-average molecular weight of the species in the eluate calculated from refractive index and light-scattering measurements. SpoIIE(590–827) protein from two different preparations was loaded onto the column at 1 mg ml^− 1^ (green and black) and 5 mg ml^− 1^ (red and blue). In all four chromatograms, the principal species present has a molecular mass of 23–27 kDa, close to the expected mass of SpoIIE(590–827) of 26,507 Da. There is evidence of higher-molecular-weight material possibly representing PP2C domain dimers. (b) Stereo ribbon representation of the structure of the SpoIIE(590–827) PP2C monomer derived from the domain-swapped dimer. The ribbon is color ramped from its N-terminus (red) to its C-terminus (magenta). The secondary structure elements are labeled. Strands β1–β3 are labeled in italics to indicate that these strands are swapped in the dimer. (c) Topology diagram of the monomeric SpoIIE–PP2C domain in which strands are shown as triangles and helices are shown as circles. The continuous and broken lines distinguish connections across the top and across the bottom of the β-sheets, respectively. The red symbols denote β-strands that are swapped in the dimer.

In earlier work, we showed clearly that SpoIIE(590–827) is monodisperse and monomeric in 50 mM Tris (pH 8.5) and 150 mM NaCl.^20^ In the light of the observation of domain-swapped SpoIIE(590–827) dimers in the crystal structure determined here, we reexamined the quaternary structure of the protein by SEC-MALLS (*s*ize-*e*xclusion *c*hromatography with *m*ulti-*a*ngle *l*aser *l*ight *s*cattering). In these experiments, samples are fractionated on a gel-filtration column and the absorbance at 280 nm and the refractive index of the eluate are monitored together with the multi-angle laser light scattering of the sample. This enables the weight-average molecular weight (*M*_w_) of species in the eluate to be calculated continuously. As shown in Fig. 4a, in 100 mM Tris (pH 8.5) and 200 mM sodium citrate, a buffer that mimics the crystallization solution, SpoIIE(590–827) at loading concentrations of 1 mg ml^− 1^ and 5 mg ml^− 1^, elutes as more than one peak from the gel-filtration column. The majority species has an *M*_w_ value close to 25,000  as would be expected for the monomer. There is evidence of higher molecular-weight minority species with shorter column retention times, and these may include domain-swapped dimers. Taken together, these data imply that SpoIIE(590–827) is a monomer under physiological conditions, which can form domain-swapped dimers under the crystallization conditions employed here.

### The structure of the monomeric PP2C domain of SpoIIE

Besides the difference in oligomeric state, the differences between a monomeric protein and a three-dimensional domain-swapped dimer are confined to the segment of polypeptide where the crossover between the domains occurs, in this case, between β3 and α1. In view of the clear evidence that SpoIIE(590–827) is a monomer in solution, we generated a model for the monomer by reassigning residues 590–630 of chain A to chain B to generate the molecule shown in Fig. 4b in which italics denote provenance from chain A. The SpoIIE(590–827) molecule has a single-domain structure consisting of 10 β-strands arranged in 2 five-stranded antiparallel β-pleated sheets. The first sheet has a strand order β1–β10–β9–β6–β7, and the second has β2–β3–β4–β5–β8 (Fig. 4b and c). The two sheets pack face-to-face against one another in the center of the molecule, with the helical segments packing against the opposing faces of each sheet to create a four-layered αββα structure (Fig. 4c). Each of the α-helical sections is contiguous and formed by segments connecting strands at the top of the molecule when viewed as in Fig. 4b. The β3–β4 segment in sheet 1 harbors helices α1 and α2, while the β9–β10 loop in sheet 2 contains α3, α4 and α5 (Fig. 4b and c). There are four crossings between the β-sheets at β1–β2, β5–β6, β7–β8 and β8–β9. The loops connecting strands at the base of the molecule β2–β3, β4–β5, β6–β7 and β8–β9 are noticeably shorter than those at the top of the molecule. The N-terminus and the C-terminus of the domain are in close proximity.

### Comparison with other PP2C structures

A search of the protein database‡ for structural similarity to the SpoIIE phosphatase domain coordinate set reveals a number of protein chains that superimpose with rmsd (*r*oot-*m*ean-*s*quare *d*eviation) values in the range 2.2–3.0 Å over 144–162 corresponding C^α^ atoms. The majority, if not all, of these structures are of protein phosphatases or the protein phosphatase domains of larger proteins.

**Fig. 5:**
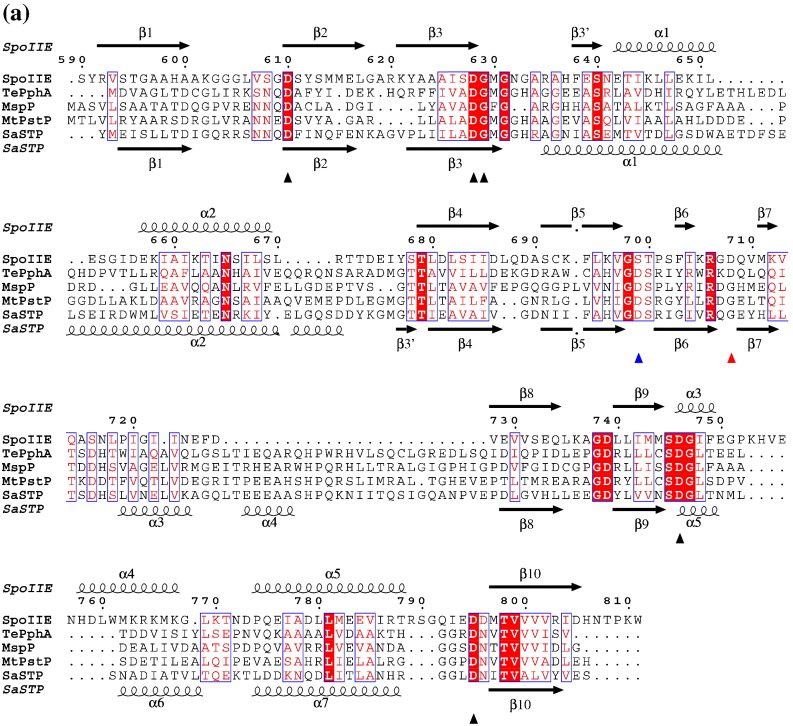

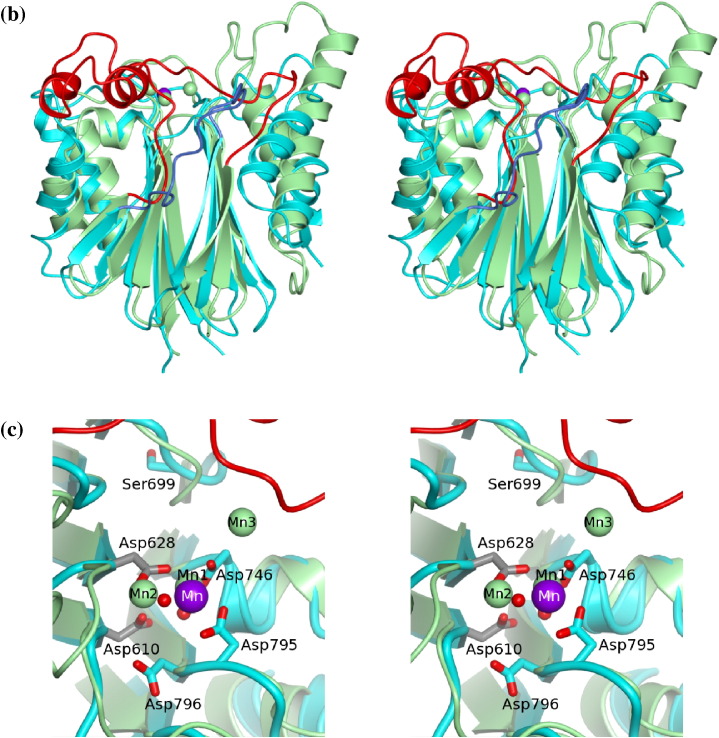
Structural and sequence comparisons among PP2C phosphatases. (a) Sequence alignment of the PP2C domain of SpoIIE with those of PphA from *T. elongatus*, MspP from *M. smegmatis*, PstP from *M. tuberculosis* and STP from *S. agalactiae*. The secondary structure elements from the structures of SpoIIE(590–827) and *Sa*STP are shown above and below the alignment, respectively. The residue numbering refers to SpoIIE(590–827). The triangular symbols refer to residues discussed in the main text. (b) Stereo overlay of the structures of SpoIIE(590–827) and *Mt*PspP represented as cyan and light-green ribbons, respectively, with Mn shown as spheres. There is a very close superposition of the β-sheet core with the principal structural deviations occurring in the loop and helical regions. The flap segment shown in blue for SpoIIE is much shorter than that in *Mt*PspP shown in red. (c) The single bound metal in SpoIIE (purple) and the three bound metals in *Mt*PspP (light green) with protein chains colored as in (b). The side chains of a cluster of Asp residues and two metal-coordinating water molecules, one of which is eclipsed by the metal, are displayed for SpoIIE. The side chain of Ser699, which takes the place of one of the Mn3-coordinating Asps in the other PP2C domains, is also shown oriented away from the metal coordination site. The differently colored carbons of Asp610 and Asp628 emphasize their origin in the domain-swapped subunit.

Among the most similar structures are those from the PP2C family, namely, human PP2Cα^21^ that possesses an additional C-terminal helical subdomain and the bacterial phosphatases PstP from *Mycobacterium tuberculosis*,^22^ MspP from *Mycobacterium smegmatis*,^23^ STP from *Streptococcus agalactiae*^24^ and tPphA from *Thermosynechococcus elongatus*^25^ (Fig. 5a and b; Fig. S3). The β-strand elements superpose closely, but there are large deviations in the β3–β4 segment, which contains α-helices 1 and 2, and in the β7–β8 segment, which is very much shorter in SpoIIE. In SpoIIE, this segment contains 15 residues in a random-coil conformation, while in the bacterial phosphatases, it is extended by 25 or so residues and includes two helical stretches (Fig. 5a and b; Fig. S3). This segment of structure is referred to as the flap region in the other bacterial PP2C phosphatases. It is proposed to control access to the active site and contribute to a substrate-binding groove. The much shorter flap in SpoIIE is inclined away from the active site and cannot obviously serve such a function.

### Manganese-binding site

Protein phosphatases of the PP2C family are manganese-dependent enzymes with the metal ions in the active sites coordinated by multiple side-chain carboxylate groups. We observed a single manganese ion at low occupancy in SpoIIE(590–827) even though the crystals were grown in the presence of MnCl_2_ (up to 50 mM). One explanation for this is that there will be competition for metal chelation between the protein and citrate (a tricarboxylate), which is present in the crystallization solutions at 0.2 M. Another is that SpoIIE(590–827) binds manganese weakly. Indeed, we were unable to detect bound manganese by atomic absorption spectroscopy following resolution, by size exclusion chromatography, of protein pre-incubated with Mn^2+^, (data not shown).

To gain structural insight into Mn^2+^ chelation in SpoIIE, we sought and obtained crystals of SpoIIE(590–827) from drops prepared from solutions in which the citrate was replaced by acetate and in which the protein was pre-incubated for 30 min with 2 mM MnCl_2_. The “citrate” and “acetate” crystals are essentially isomorphous, and a domain-swapped dimer is again observed (Table 1). In the latter structure, we observed a single high-occupancy manganese (Mn^2+^) ion in the active site. Its identity was confirmed by anomalous difference maps that revealed a peak, 6σ above the mean (Fig. S4), in the active sites of both molecules in the asymmetric unit, coincident with the positions of Mn^2+^ in the refined coordinate set. This single Mn^2+^ per protein chain in SpoIIE contrasts with the two metal ions found in human PP2Cα^21^ and the three observed in the bacterial protein phosphatases PstP, MspP, STP and tPphA.^22,24–26^ The manganese ion in each active site of SpoIIE is coordinated by the side-chain carboxylates of Asp628, Asp746 and Asp795 with the Asp628 ligand provided by the partner subunit in the domain-swapped dimer. The three metal ions in PstP, MspP, STP and tPphA are almost exactly superposed following an overlap of the structures based on protein C^α^ positions. In this comparison, the single metal ion in the SpoIIE structure superimposes closely with the first of the manganese ions, Mn1, of the bacterial phosphatases as shown for *Mt*PstP in Fig. 5c. The octahedral coordination and the coordinating ligands (contributed by three carboxylates and two water molecules) are conserved in SpoIIE.

There would appear to be capacity in SpoIIE to coordinate a second Mn species, equivalent to Mn2 in the other bacterial phosphatases, since the corresponding protein ligands Asp628, Gly629 and Asp610 are conserved and appropriately situated (Fig. 5a and c). One explanation for the absence of Mn2 is that SpoIIE does not bind a second metal ion or that it does so with lower affinity. Another possibility is that Mn2 coordination is compromised by the domain-swapping event. All three of the putative Mn2-coordinating residues are in the amino-terminal subdomain that precedes the site of domain swapping. Asp628 and Gly629 are immediately adjacent to the disordered segment at this site; however, the *B*-values of these residues are not significantly higher than the average residue temperature factors overall. The binding of Mn2 does not appear to be restricted by crystal packing, as this part of the structure does not participate in lattice interactions.

There does not however appear to be capacity to bind a third Mn^2+^, equivalent to Mn3, since only one of the coordinating protein ligands is conserved, this being Asp746 whose equivalent bridges the Mn1 and Mn3 metal ions in the other bacterial phosphatase structures. It had been expected from sequence comparisons that Asp708 in SpoIIE, denoted by the red triangle in Fig. 5a, would correspond to Asp118 (blue triangle) in PstP and STP; however, the structure shows that this is not the case and that Asp708 is at the opposite end of strand β6 on a face of the molecule distal to the active site. The side chain of Ser699, which does correspond to Asp708, is oriented away from the metal coordination site (Fig. 5c) and plays a structural role in the β5–β6 loop, its hydroxyl forming polar interactions with the main-chain amino and carbonyl groups of residues 697 and 700, respectively, and with the side-chain amino group of Lys696. Meanwhile, a third Mn3-coordinating ligand in PstP (Ser160) is not conserved, occurring as it does, in the extended β7–β8 region that is curtailed in SpoIIE. In STP and tPphA, polar residues replace Ser160. In the case of STP, Asn160 stabilizes a metal-coordinating water molecule.

Otherwise, the alignment of the sequences of *Mt*PstP, MspP, *Sa*STP, *Te*PphA and *Bs*SpoIIE shows that conserved residues are largely confined to the metal coordination sphere and its neighbors. A notable exception is the Arg706 and Asp739 pair that forms a salt bridge.

## Discussion

Reversible protein phosphorylation by protein kinases and protein phosphatases is a major form of signaling and regulation in living cells. SpoIIE belongs to the metal-dependent protein phosphatase (PPM) family exemplified by human PP2C. In contrast to the PPP family enzymes that function in combination with different regulatory subunits, the PPM family members have additional domains that help to determine substrate specificity. In SpoIIE, the PP2C domain is preceded by 590 residues that form at least two functional domains (Fig. 1c). The amino-terminal 320 or so residues form a substantial transmembrane domain (domain I), while the central 270 or so residues form a domain that is implicated in binding to the cell division protein FtsZ and in sporulation septum formation (domain II). Interaction of the upstream domains I and II, with the PP2C domain III, therefore provides a means of coupling σ^F^ activation to asymmetric septation.

The pathway of σ^F^ activation begins with the dephosphorylation of SpoIIAA∼ P by SpoIIE and culminates in the displacement of σ^F^ from the complex with SpoIIAB by SpoIIAA. The establishment of differential gene expression in sporulation depends on the confinement of σ^F^ activity to the forespore, which implies that either the phosphatase activity of SpoIIE is held in abeyance until the asymmetric septum is completed or dephosphorylated SpoIIAA, once formed, is not immediately available to induce the release of σ^F^ from the complex with the anti-sigma factor. Different models for how SpoIIE brings about activation of σ^F^ in the forespore, but not in the mother cell, have been proposed. The models variously invoke the volume difference between the compartments, the preferential localization of SpoIIE to the forespore face of the septum and/or transient genetic asymmetry.^27–29^

### Regulation of phosphatase activity

**Fig. 6:**
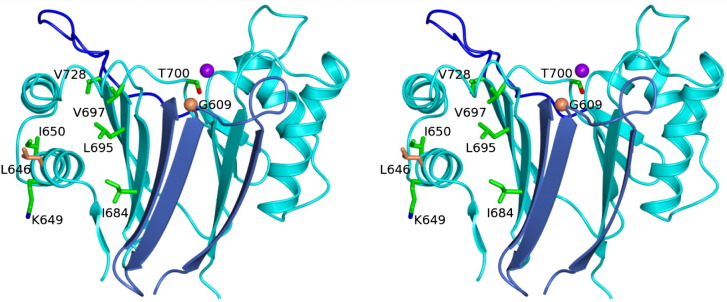
Mutational mapping. The SpoIIE chains are shown in ribbon format with cyan and light-blue coloring indicating the different chains of the domain-swapped dimer. The dark-blue color represents the flap region. In cylinder representation with atoms colored according to type are side chains of residues identified in genetic studies of SpoIIE function. Side chains with green carbon atoms indicate residues whose mutation suppresses the effect of *spoIIE48* or the Gln483-to-Ala mutation. The coral-colored Leu646 and Gly609 (shown as a C^α^ sphere) are the sites of mutation in *spoIIE64* and *spoIIE71*, respectively.

The role of SpoIIE in sporulation has been studied for many years, and a rich catalogue of *spoIIE* mutants has been previously mapped and characterized. Some of the mutations map to the phosphatase domain (domain III). Among these, the best-characterized mutations are *spoIIE64* (Leu646Phe), *spoIIE71* (Gly609Asp) and *spoIIE20* (Gly698 to STOP).^30^ These three mutations prevent σ^F^ activation, and cells harboring these alleles cannot sporulate. In these strains, the phosphatase activity is impaired either by the deletion of a large region of the phosphatase domain (*spoIIE20*) or through the substitution of a residue that is crucial for the phosphatase function of the domain (*spoIIE71*). Gly609 on the β1–β2 loop is integral to the active site (Fig. 6), and its mutation to Asp would be expected to alter the conformation of neighboring residues such as Asp610. Asp610 is a would-be ligand of the putative Mn2. Gly609 is invariant in the sequences of SpoIIE orthologues from *Bacilli* and *Clostridiae* shown in Fig. S5. The site of the substitution associated with *spoIIE64* is residue 646. Leu646 is remote from the active site, situated as it is, in the middle of helix α1 (Fig. 6). Leu646 is conserved (as Leu/Ile) in SpoIIE orthologues and in the bacterial phosphatases shown in Fig. 5a. Substitution of Leu by Phe in many circumstances represents a relatively conservative change. As discussed below, a cluster of mutations that alter the function of SpoIIE maps to helix α1 and its environs, and we propose that this region of the structure is important in the regulation of enzyme activity. Cells harboring the *spoIIE64* and *spoIIE71* alleles form asymmetric septa morphologically identical with those formed by wild-type cells.^10^ In contrast, for the *spoIIE20* mutant strain, not only is the phosphatase activity affected but so too is the nature of the polar septum that is formed. As observed in a number of strains harboring mutations in domain II of SpoIIE, the asymmetric septum is thicker and resembles a vegetative septum.^10,11^ This indicates that domain III is important not only for its phosphatase activity but also for proper sporulation septum formation, probably through its interactions with domain II.

Further support for the idea that there is domain II regulation of the PP2C phosphatase domain activity comes from a further well-studied mutant *spoIIE48* (Ser361Phe)^30^ in which both phosphatase activity and sporulation septum formation are impaired. A similar phenotype was observed following mutation of Gln483 to Ala.^31^ A number of intragenic suppressors of these mutations have been identified, and a subset was shown to map within the PP2C domain. The mutations Lys649Thr, Ile650Leu, Ile684Val, Leu695Trp, Thr700Pro and Val728Met suppress the effects of the Ser361Phe mutation, while Val697Ala is a suppressor of both Ser361Phe and Gln483Ala mutations.^31,32^ Mapping of the sites of these suppressor mutations onto the structure of the PP2C domain reveals that four of the residues, Ile684, Leu695, Val697 and Val728, are on the same face of a β-sheet that packs against helix α1 on which another two of these residues Lys649 and Ile650 reside (Fig. 6). The remaining suppressor Thr700Pro, with its introduction of a proline residue into a loop adjacent to the β-sheet, might also lead to a similar perturbation of this region of the structure. A speculative interpretation of these data is that mutations such as *spoIIE48* strengthen an inhibitory interaction of domain II with the PP2C domain involving an interface that encompasses helix α1. Mutations of residues elsewhere on this surface, or of residues determining the juxtaposition of this surface and the rest of the protein, would be expected to weaken these intramolecular interactions and restore activity to near wild-type levels.

Additional mutants have been isolated in *spoIIE* (*spoIIEV697A*, *spoIIE19* and *spoIIE26*), which allow σ^F^ to be activated independently of septum formation.^33^ Interestingly, *spoIIEVal697Ala*, which maps to domain III, was identified independently as a suppressor of *spoIIE48*, which maps to domain II. In contrast, the alterations encoded by *spoIIE19* (Gln344Pro) and *spoIIE26* (Gly334Arg) map to the boundary between the transmembrane domain and domain II. The fact that these mutants are otherwise fully functional suggests the presence of regulatory sites in domain II and domain III of SpoIIE that tightly control the phosphatase activity in response to asymmetric septation.^33^ The structure of the PP2C domain of SpoIIE determined here suggests that the active-site region is accessible to phosphorylated SpoIIAA, implying that this domain is intrinsically active. It seems likely therefore that regulation would be exerted through inhibition of activity in the intact protein possibly by occlusion of the active site by domain II in concert with other components of the cell division machinery.

### Manganese binding and mechanism

The most striking feature of the structure of the phosphatase domain of SpoIIE is the presence of a single manganese ion in the active site, contrasting with the two in human PP2C and the three in the bacterial PPM homologues whose structures are known. It is unclear whether this represents a fundamental difference between SpoIIE and the other PP2C type phosphatases, although it is notable that the sequence motifs characterizing these enzymes^34^ are less well conserved in SpoIIE (Fig. 5a). SpoIIE with the sequence GLVSGD^610^ lacks the arginine residue of the RxxxxD motif. This arginine forms an ion pair with a phosphate ion in the human PP2C structure that is thought to mimic the phosphate of the phosphoserine substrate.^21^ The DGMGN^632^ sequence lacks the second glycine of the DGxxG motif, and finally, the QIEDD^796^ sequence lacks the glycine and asparagine residues of the GxxDN motif.

The hierarchy of manganese binding to the three metal chelation sites in the bacterial phosphatases has been studied by isothermal titration calorimetry in *Mt*PstP and MspP.^23^ The three metal-binding sites have distinct affinities that are in the nanomolar and micromolar ranges for Mn1 and Mn2, respectively, while the affinity for Mn3 is much lower. The presence or absence of the third “volatile” Mn^2+^ correlates with different conformations of the flap segment, which is implicated in enzyme substrate interactions. The flap is much less substantial in SpoIIE, an observation that correlates with the absence of the third metal (M3) binding site, although M2 (and even M1) is apparently also “volatile” in this system. The role of the third metal has been investigated in tPphA and human PP2C by mutagenesis of the coordinating Asp119 and Asp146, respectively. Substitution of the Asp with residues incapable of metal coordination led to loss of M3 binding in tPphA and a large decrease in activity in both enzymes, leading the authors to propose a catalytic role for M3 in activating a water molecule that protonates the leaving group.^35^ This Asp is conserved in numerous PP2C proteins, but not in SpoIIE where it is replaced by Ser699 (Fig. 5a). This hints at alternative aspects to the mechanism in the SpoIIE-catalyzed dephosphorylation of SpoIIAA∼ phosphate.

### Implications for SpoIIAA interactions

The understanding of substrate recognition by protein serine phosphatases (PSPs) is very limited, and in many instances, the physiological substrates are unknown.^34^ There are a handful of structures of PSPs bound to substrate peptides, but there is no structural information available for a PSP bound to a phosphoprotein. The crystal structure of SpoIIAA∼ P, the substrate of SpoIIE, from *B. sphaericus* is known (Fig. 1b). The site of phosphorylation in SpoIIAA, Ser57 (Ser58 in SpoIIAA from *B. subtilis*), is exposed on the surface, and attachment of the phosphate is not associated with any significant conformational change. Instead, it seems that the bulk and charge of the additional phosphate group are sufficient to flag the change in status of the protein. Besides the covalent linkage to this side chain's hydroxyl, there are no further intramolecular interactions of the phosphate group (Fig. 1b).

To explore possible interactions of the SpoIIE PP2C domain with SpoIIAA∼ P, we performed a simple rigid-body docking experiment. First, we overlaid the SpoIIE(590–827) structure onto that of human PP2Cα^21^ that contains a phosphate group in the active site. We next superposed this phosphate group and the phosphate of the Ser57 phosphate in the structure of SpoIIAA∼ P from *B. sphaericus*.^14^ There are three ways in which the phosphate oxygens can be exactly superposed, two of which lead to extensive steric clashes. As shown in Fig. S6, the third orientation generates a model in which there are no contacts between the SpoIIE and the SpoIIAA∼ P. We next carried out steps of rigid-body energy minimization, but we were not able to find a satisfactory candidate model of the complex. One obvious explanation is that conformational changes in SpoIIE accompany SpoIIAA∼ P binding. The SpoIIE structure around the Mn^2+^-containing active site consists of multiple loop regions, some of which are disordered in the crystal. Another explanation is that the crystal structure of the *B. sphaericus* SpoIIAA∼ P does not accurately represent the structure of SpoIIAA∼ P from *B. subtilis*, which is a much better substrate for SpoIIE (Fig. 2). Many of the residues contributing to the surface of SpoIIAA∼ P surrounding the phosphorylated serine differ between the two orthologues.

### Concluding remarks

The structure of the phosphatase domain of SpoIIE determined here provides a useful framework for interpreting genetic data on this well-studied protein and for formulating ideas about its mechanism of action. A fuller understanding of how completion of the asymmetric cell division septum triggers σ^F^ activation requires structures of larger SpoIIE fragments encompassing the central FtsZ binding domain and structural data on complexes of SpoIIE with FtsZ and SpoIIAA∼ P.

SpoIIE is very likely to form oligomers *in vivo*, and it has been shown to multimerize *in vitro*.^18^ In our crystal structure, we observed domain-swapped dimers. Domain swapping is a *bona fide* mechanism of homodimer and homomultimer assembly,^36^ and indeed, domain swapping has been observed in the crystal structures of a number of proteins associated with bacterial cell division and sporulation including Spo0A,^37^ SpoVT,^38^ FtsZ^39^ and MinC (Protein Data Bank entry 3GHF). However, it is often difficult to demonstrate the physiological significance of domain swapping, and in many instances, including Spo0A,^40^ it has been shown to be an artifact of protein handling. Solution studies show that the PP2C domain of SpoIIE is monomeric, indicating that dimerization/oligomerization is associated with the upstream domains in this protein.

Five PSPs have been identified in *B. subtilis* all belonging to the PP2C class. PrpC is thought to be involved in coordinating cell wall expansion and cell growth, and its substrates include the translation factor EF-Tu and the circularly permuted GTPase, CpgA.^41,42^ Like SpoIIE, the phosphatases RsbP, RsbU and RsbX regulate the activity of an alternative sigma factor, in this case, the environmental stress response sigma factor σ^B^. The structure of an RsbT-binding fragment of RsbU revealed a dimer-forming four-helix bundle domain.^43^ RsbU dephosphorylates the SpoIIAA-like protein RsbV, and its activity is stimulated by interaction with RsbT. The structure of the C-terminal phosphatase domain is not yet reported, but with such obvious parallels running through the two systems, it will be interesting to compare the structural basis of phosphatase domain activation in SpoIIE and RsbU.

## Materials and Methods

### Expression and purification

A DNA fragment encoding a soluble domain of the *B. subtilis* SpoIIE phosphatase (residues 590–827) was amplified by PCR, using the primers E590-F and E827-R (Table S1), and was inserted into the expression vector pET-YSBLIC3C^44^ using ligation-independent cloning. The resulting recombinant plasmid directs the expression of a SpoIIE fragment fused to a human rhinovirus 3C protease-cleavable N-terminal His_6_ tag. The plasmid was introduced into *E. coli* BL21 (DE3) and the methionine auxotroph *E. coli* B834 (DE3) for overexpression of the SpoIIE phosphatase domain in native and SeMet substituted forms, respectively.

Overnight cultures were used to inoculate either 500 ml of LB medium for the production of the native protein or 500 ml of minimal media containing SeMet for the production of the derivative protein. In each case, the media were supplemented with 30 μg ml^− 1^ kanamycin. For overexpression, cells were grown to an OD_600_ of 0.6–0.7 at 37 °C, and recombinant protein production was induced by the addition of 1 mM isopropyl-β-d-thiogalactopyranoside. For native protein production, this was followed by growth at 16 °C overnight. For production of the SeMet-labeled protein, the cells were subsequently grown at 37 °C for 4 h. The purification procedure was similar for both proteins. The cells were harvested by centrifugation and resuspended in 20 mM sodium phosphate, 0.5 M NaCl (BufferA) and 10 mM imidazole (pH 7.5) in the presence of a protease inhibitor cocktail (Roche). The cells were lysed by sonication, and the lysate was clarified by centrifugation. Supernatant containing the target protein was applied to a HiTrap Ni-affinity column (GE Healthcare), and bound protein was eluted with an ascending concentration gradient (10 mM–0.5 M) of imidazole in Buffer A. This step was followed by gel-filtration chromatography on a Superdex S200 column, equilibrated with 20 mM Tris–HCl (pH 8.0), 100 mM NaCl and 5 mM DTT. The amino-terminal purification tag was removed by treatment with 3C protease, and after a second passage of the proteins through the Ni column and an additional gel-filtration step, we obtained protein samples of 98–99% purity as judged by Coomassie blue staining of sodium dodecyl sulfate polyacrylamide gels.

SpoIIAA was prepared from *E. coli* cultures expressing *spoIIAA*, and SpoIIAA∼ P was prepared from cells co-expressing *spoIIAA* and *spoIIAB* as described previously.^14^ For this purpose, the appropriate coding sequences were amplified by PCR using combinations of the primers AA-F, AA-R and AAAB-R (Table S1) and were introduced into pET-YSBLIC3C^44^ by ligation-independent cloning. The resulting plasmids were prepared from *E. coli* NovaBlue GigaSingles and introduced into competent *E. coli* B834 (DE3) for expression and co-expression of *spoIIAA* or *spoIIAA*-*spoIIAB*, respectively, by isopropyl-β-d-thiogalactopyranoside induction. In each case, the SpoIIAA protein is fused to an N-terminal 3C-protease-cleavable His_6_ tag. Following cell lysis, purification of SpoIIAA and SpoIIAA∼ P was achieved by nickel chelation chromatography, cleavage with 3C protease, passage through a second nickel-chelating column and, finally, fractionation on a gel-filtration column equilibrated in 20 mM Tris and 150 mM NaCl (pH 8.0).

### Crystallization, data collection and structure solution

For crystallization experiments, the purified SpoIIE(590–827) protein was concentrated to 25–50 mg ml^− 1^ in 20 mM Tris–HCl (pH 8.0) and 100 mM NaCl. After concentration, the sample of SpoIIE(590–827) was clarified by centrifugation to remove precipitated material.

Crystals were grown in hanging drops by mixing the concentrated protein with a reservoir solution in a 1:1 ratio. For crystallization of SpoIIE(590–827), the well solution contained 14–15% polyethylene glycol 400, 0.1 M Tris–HCl (pH 8.5), 0.2 M sodium citrate and 0–50 mM MnCl_2_. For the SeMet form of SpoIIE(590–827), a third well solution containing 1.4–1.6 M sodium acetate and 0.1 M sodium cacodylate (pH 6.5) was optimal. The best-diffracting crystals of SpoIIE(590–827) were obtained at 37 °C. Crystals were frozen in a nitrogen gas stream after soaking in mother liquor supplemented with 25% (v/v) glycerol.

The SpoIIE(590–827) crystals and the SeMet derivative crystals belonged to space group *P*6_1_22, with unit cell parameters *a* = *b* = 87.6 Å, *c* = 321.6 Å, α = β = 90° and γ = 120°, and contained two molecules in the asymmetric unit. X-ray diffraction data from the native protein crystals were collected to 2.6 Å resolution on beamline ID14-2 of the European Synchrotron Radiation Facility (ESRF), Grenoble, and a three-wavelength data set from the SeMet crystals suitable for MAD phasing was collected to 3.05 Å resolution on beamline I02 at the Diamond Light Source (Harwell). All data were processed using the HKL2000 package.^45^ Details of diffraction data and processing statistics are given in Table 1.

The SpoIIE(590–827) structure was solved using MAD phasing to 3.5 Å, and subsequent phase extension was carried out against 2.6 Å resolution native data. The two molecules in the asymmetric unit provided a total of 22 selenium sites for use in phasing. Of these selenium sites, 17 were located using a Patterson search method implemented in the program SHELXD.^46^ The electron density maps were of good enough quality for us to generate a starting model in the program Coot,^47^ the Se sites serving as useful markers in this process. The resulting model was used for refinement against the native data using restrained maximum-likelihood methods as implemented in REFMAC^48^ with a bulk solvent correction applied and with TLS (*t*ranslation, *l*ibration and *s*crew-rotation displacement) restraints. Cycles of refinement interspersed with sessions of manual refitting led to a final *R*-value of 0.22 (*R*_free_ = 0.28) using all data in the resolution range 10.0–2.6 Å. The refinement statistics are given in Table 1.

SpoIIE(590–827) crystals containing Mn were obtained from drops prepared from well solutions containing 0.9–1.2 M sodium acetate and 0.1 M Hepes, pH 7.5. The protein at 28 mg ml^− 1^ was pre-incubated with 1–2 mM MnCl_2_ for 30 min . The crystals were optimized by seeding under similar conditions, but with 1–2 mM MnCl_2_ added to the well solution. Interestingly, the crystals grown with 10–50 mM MnCl_2_ in the well solution without pre-incubation of the protein with the metal ions were found to be free of manganese.

### Phosphatase assay

SpoIIAA∼phosphate phosphatase assays of SpoIIE(590–827) were performed with recombinant *B. subtilis* SpoIIAA. A series of reaction mixes containing 1 μl of 0.05 mg ml^− 1^ SpoIIE(590–827) and 5 μl of 10 mg ml^− 1^ SpoIIAA∼ P (giving a SpoIIE:SpoIIAA∼ P molar ratio of 1:2000) in a volume of 10 μl of 20 mM Tris (pH 8.0), 100 mM NaCl and 20 mM MnCl_2_ was set up. The reactions proceeded for between 15 and 180 min  at room temperature at which point 10 μl of loading dye was added to the sample, and the reaction products were resolved by 7.5% non-denaturing polyacrylamide gel electrophoresis. Gels were run at 100 V for 140 min  at 4 °C, and the proteins were visualized by staining with Coomassie blue dye.

### Size-exclusion chromatography with multi-angle laser light scattering

For determination of the oligomeric state of the proteins, the SpoIIE fragment was analyzed using an HPLC-MALLS apparatus as described previously.^49^ We loaded 0.1-ml samples of protein at ∼ 1–5 mg ml^− 1^ onto a Superdex 200 10/300 gel-filtration column equilibrated at 0.5 ml min^− 1^ with a mobile phase consisting of 10 mM Tris–HCl and 200 mM sodium citrate at pH 8.5. The eluate was passed successively though an SPD20A UV/Vis detector, a Wyatt Dawn HELEOS-II 18-angle light-scattering detector and a Wyatt Optilab rEX refractive index monitor with the system driven by a Shimadzu HPLC system comprising an LC-20AD pump. The data were processed, and molecular masses were calculated using the Astra V software (Wyatt).

### Accession numbers

The coordinates and structure factors for SpoIIE(590–827) and the manganese-coordinated SpoIIE(590–827) have been deposited in the Protein Data Bank with accession codes 3T91 and 3T9Q, respectively.

## Figures and Tables

**Table 1:** X-ray data collection and refinement statistics

Crystal	SpoIIE_PD 590–827 (aldohexose)_	SpoIIE_PD 590–827 (Mn)_	SpoIIE_PD 590–827 (SeMet peak)_	SpoIIE_PD 590–827 (SeMet inflection)_	SpoIIE_PD 590–827 (SeMet remote)_
*Data collection*					
X-ray source	ID14-2, ESRF	ID14-4, ESRF	I02, DLS	I02, DLS	I02, DLS
Wavelength (Å)	0.93300	0.93930	0.97950	0.97970	0.9755
Resolution range (Å)	50.00–2.6	50.00–2.76	50.00–3.5	50.00–3.5	50.00–3.5
Space group	*P*6_1_22	*P*6_1_22	*P*6_1_22	*P*6_1_22	*P*6_1_22
Unit cell parameters					
*a* = *b*, *c* (Å)	87.6, 321.6	86.0, 322.5	86.6, 316.9	86.6, 317.0	86.6, 317.0
α = β, γ (°)	90, 120	90, 120	90, 120	90, 120	90, 120
Number of unique reflections, overall/outer shell^a^	23,618/1129	19,173/871	9693/459	9,64/460	9680/460
Completeness (%), overall/outer shell^a^	99.9/98.3	99.5/94.8	100.0/100.0	99.8/100.0	99.8/100.0
Redundancy, overall/outer shell^a^	7.3/4.3	16.8/16.1	39.5/40.3.2	19.9/20.5	19.9/20.6
*I*/σ(*I*), overall/outer shell^a^	30.4/1.2	64.2/7.7	37.0.9/8.5	35.5/6.7	37.3/6.1
*R*_merge_^b^ (%), overall/outer shell^a^	6.5/89.0	6.6/39.4	14.9/52.6	12.7/47.0	12.6/48.1

*Refinement and model statistics*					
Resolution range (Å)	43.81–2.6	43.58–2.76			
*R*-factor^c^ (*R*_free_^d^)	0.23 (0.29)	0.22 (0.30)			
Reflections (working/free)	22,278/1264	18,015/979			
Outer-shell^e^*R*-factor^c^ (*R*_free_^d^)	0.39 (0.38)	0.27 (0.44)			
Outer-shell reflections (working/free)	1590/72	1273/73			
Molecules per asymmetric unit	2	2			
Number of protein non-hydrogen atoms	3393	3425			
Number of water and small-molecule atoms	161	37			
rmsd from target^f^					
Bond lengths (Å)	0.008	0.020			
Bond angles (°)	1.335	2.001			
Average *B*-factor (Å^2^)	41.2	67.8			
Ramachandran plot^g^	90.2/9.0/0.8/0.0	79.6/17.6/1.5/1.3			

a The outer shell corresponds to 2.64–2.60 Å [SpoIIE_PD 590–827 (aldohexose__)_], 2.81–2.76 Å [SpoIIE_PD 590–827 (Mn__)_] and 3.56–3.5 Å [SpoIIE_PD 590–827 (SeMet)_].

b *R*_merge_ = ∑*_hkl_*∑*_i_*|*I_i_* − 〈*I*〉|*/*∑*_hkl_*∑*_i_*〈*I*〉, where *I*_*i*_ is the intensity of the *i*th measurement of a reflection with indexes *hkl*, and 〈*I*〉 is the statistically weighted average reflection intensity.

c *R*-factor = ∑||*F*_o_| − |*F*_c_||*/*∑|*F*_o_|, where *F*_o_ and *F*_c_ are the observed and calculated structure factor amplitudes, respectively.

d *R*_free_ is the *R*-factor calculated with 5% of the reflections chosen at random and omitted from refinement.

e Outer shell for refinement corresponds to 2.600–2.663 Å (aldohexose), 2.833–2.762.

f rmsd of bond lengths and bond angles from ideal geometry.

g Percentage of residues in most favored/additionally allowed/generously allowed/disallowed regions of the Ramachandran plot.

## Footnotes

‡ using the resources available at www.ebi.ac.uk.
