# Supplementary material for: Structure of the Phosphatase Domain of the Cell Fate Determinant SpoIIE from Bacillus subtilis
Source: J Mol Biol. 2012 Jan 13;415(2):343–58. doi: 10.1016/j.jmb.2011.11.017 (PMC3517971; doi:10.1016/j.jmb.2011.11.017)

Supplementary Material.

Contents:

Table S1.	Oligonucleotide primers used in this work	1
Supplementary Figure Legends		1
Figure S1.	Assays of Phosphatase Activity	3
Figure S2	Topology diagram of the domain-swapped dimer	3
Figure S3	PP2C domain structure comparisons	4
Figure S4	Anomalous difference density for Manganese	4
Figure S5.	Alignment of sequences of SpoIIE orthologues	5
Figure S6	Model of the SpoIIE-SpoIIAA~P complex	5

Table S1. Oligonucleotide primers used in this work

E590-F	5'-CCAGGGACCAGCAATGTCATACAGGGTGTCCACGGGAGCTGCTCAC-3'
E827-R	5' GAGGAGAAGGCGCGTTATGAAATTTCTTGTTTGTTTTGAAAGATTGCCG-GAAC-3'
AA-F	5'-CCAGGGACCAGCAATGAGCCTTGGAATTGACATGAATG-3'
AA-R	5'-GAGGAGAAGGCGCGTCATGATGCCACCCCCAGTGT -3'
AAAB-R	5'-GAGGAGAAGGCGCGTTAATTACAAAGCGCTTTGCTTTTTG-3'

Supplementary Figure Legends

Figure S1. Coomassie blue stained non-denaturing polyacrylamide gel of SpoIIE(590-827) phosphatase activity. SpoIIE(590-827) and SpoIIAA-P were mixed (a molar ratio of 1 : 100) in the presence and absence of metals/metal chelators, incubated for 45 minutes, and the samples were loaded onto a 7.5 % polyacrylamide gel. Electrophoresis was performed at 4°C for 140 min at 100 V. Lane 1: 10 µg SpoIIAA; Lane 2: 10 µg SpoIIAA-P; Lane 3: 8 µg SpoIIE(590-827). Lane 4: reaction in the absence of $MnCl_2$; Lane 5: reaction in the presence

of 20 mM $MnCl_2$; Lane 6: reaction in the presence of 20 mM $MgCl_2$; Lane 7 reaction in the presence of sodium citrate (0.2 M) and 20 mM $MnCl_2$.

Figure S2. Topology diagram of the domain-swapped SpoIIE-PP2C dimer in which strands are shown as triangles and helices as circles. The chains are coloured blue and red and breaks in the chain are indicted by pairs of cross bars. The solid and dotted lines distinguish connections across the top and across the bottom of the β-sheets respectively.

Figure S3. Superposition of human PP2C (PDB code 1aq6; magenta) with those of tPphA from *T. elongatus* (2j82; green), MspP from *M. smegmatis* (2v06; light green) PstP from *M. tuberculosis* (1txo; pink) and STP from *S. agalactiae* (2pk0; red) and the PP2C domain of SpoIIE (cyan). The chains are shown in stereo as worms and with metal ions as spheres and coloured by chain. The A chains of each coordinate set were superposed using the SSM superpose routine in CCP4mg. The close superposition of the structures across their β-sheets (centre) constrasts with the striking variability in the flap region (top front). The extra helical region of human PP2C is on the left.

Figure S4. Anomalous difference Fourier map calculated with phases from the refined structure and measured anomalous differences from the $SpoIIEPD_{590\text{-}827(Mn)}$ data set (Table 1). The map is contoured at 6σ above the mean and displayed over the entire model clearly confirming the presence of the scattering from the Mn ions. The structure is shown as ribbons coloured by chain with the hexose in ball-and-stick format. Two manganese ions from the refined model are shown as spheres in the anomalous difference density.

Figure S5. Alignment of the sequences of the phosphatase domains of SpoIIE orthologues from endospore-forming bacteria with the secondary structure elements of the *Bsu* SpoIIE phosphatase domain overlaid. The sequences aligned are BACSU, *B. subtilis*; BACAN, *B. anthracis* (strain A0248); GEOBA, *Geobacillus sp.* Y412MC52; BACC1, *B. cereus* (strain ATCC 10987); BACHD, *B. halodurans*; BACCR, *B. cereus* (strain ATCC 14579 / DSM 31); CLOB1, *Clostridium botulinum* (strain ATCC 19397 / Type A); CLOD6, *C. difficile* (strain 630).

Figure S6. A model of the interaction of SpoIIE (ribbon with Mn^{2+} and coordinating residues shown) and SpoIIAA~P (green ribbon with the phosphorylated Ser57 shown. The model was generated by (i) superimposing the SpoIIE coordinates onto those of human PP2Cα using the SSM superpose routines in CCP4mg and (ii) superimposing the phosphate atoms of the serine-phosphate in the structure of SpoIIAA~P from *B. sphaericus* (green) and the phosphate ion in the active site of human PP2Cα.

Figure S1

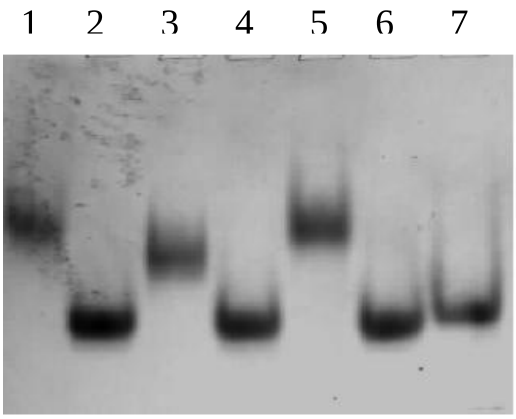

Figure S2

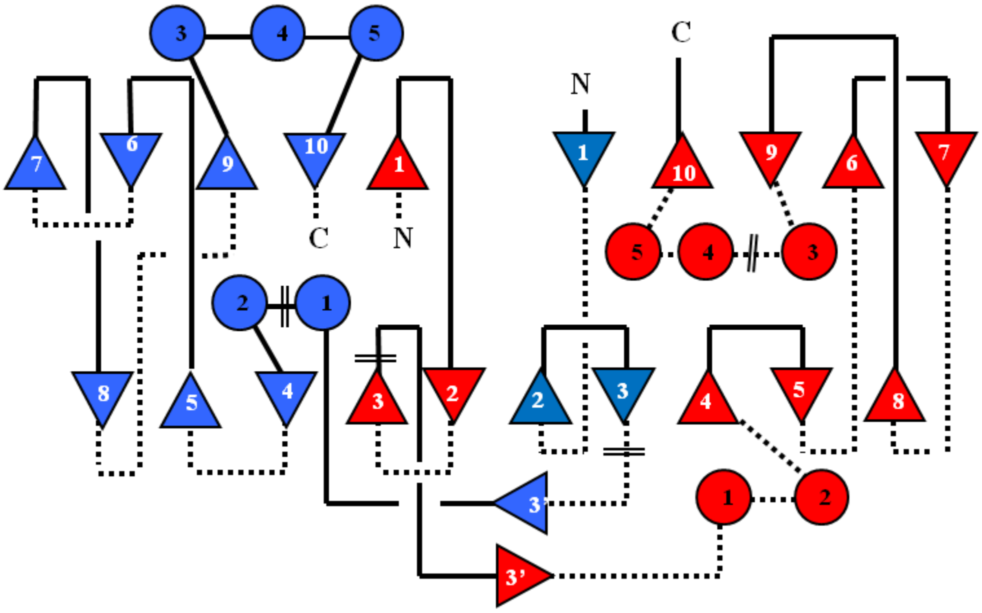

Figure S3

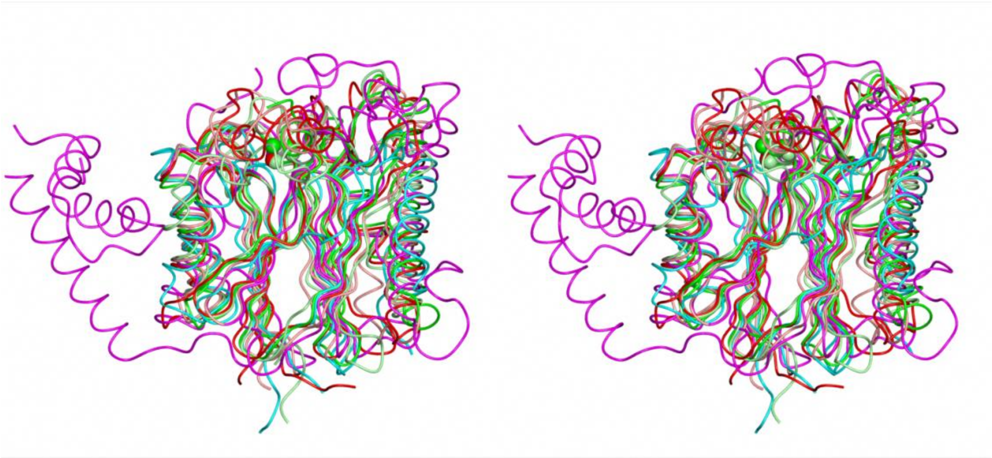

Figure S4

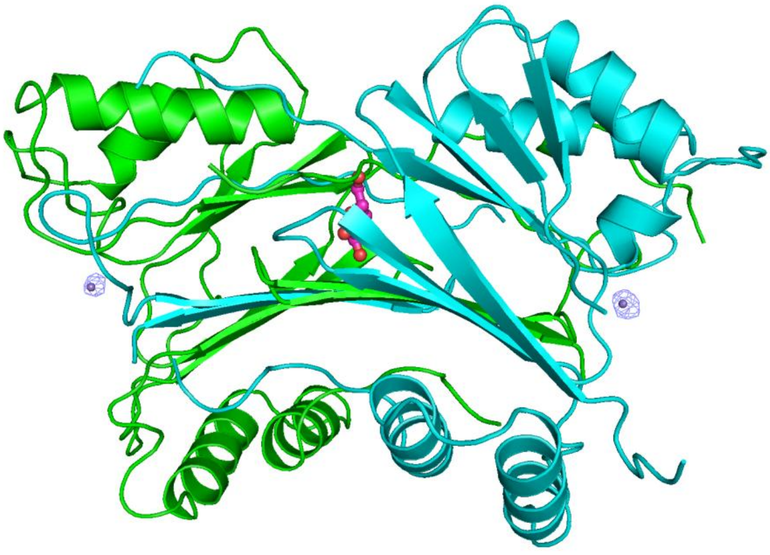

Figure S5.

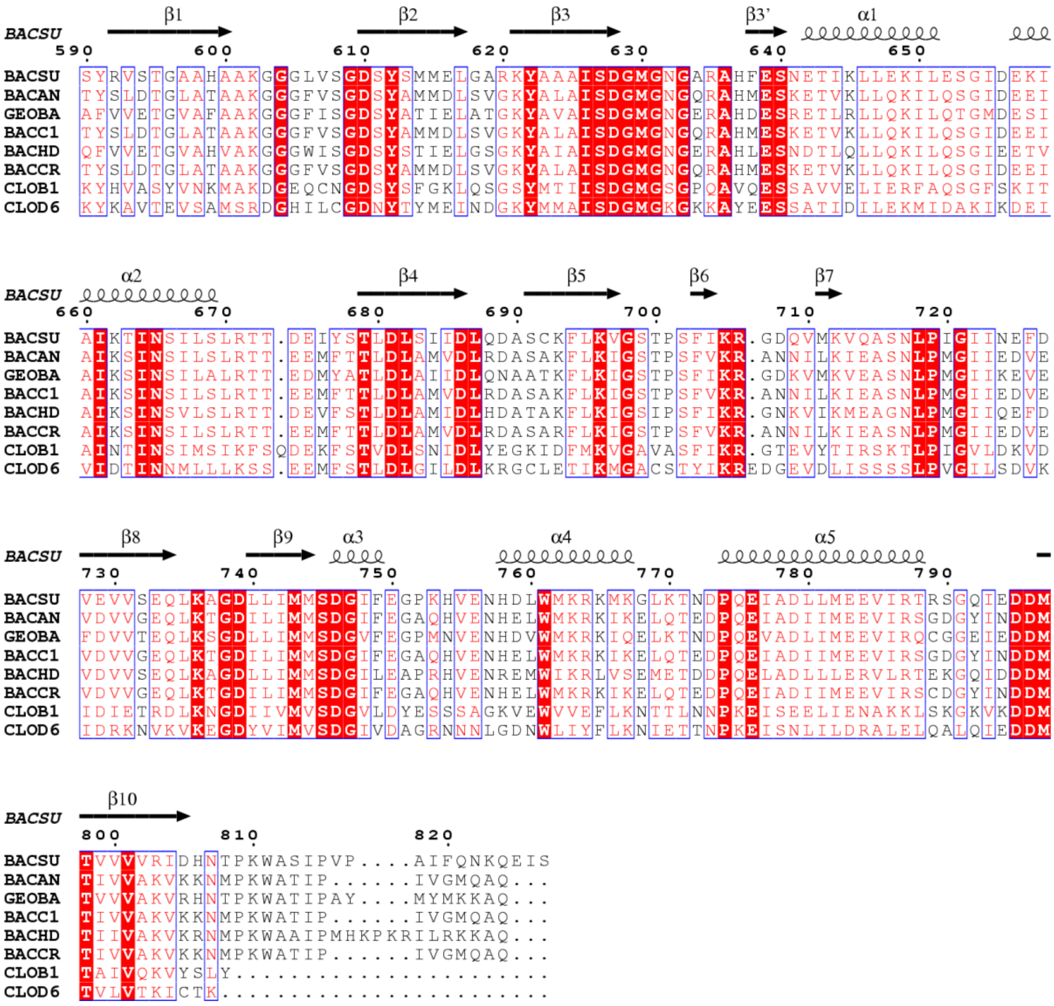

Figure S6.

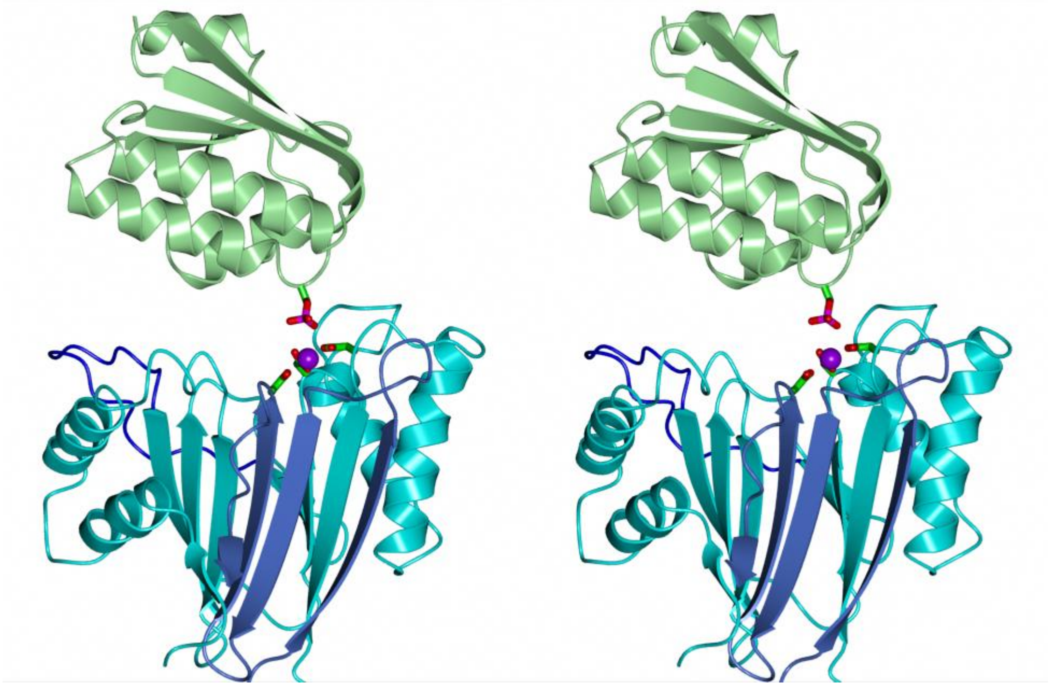

Supplement: Supplementary file 1 — Supplementary materials [file mmc1.pdf]
